# Epigenetic modifications in chronic rhinosinusitis with and without nasal polyps

**DOI:** 10.3389/fgene.2022.1089647

**Published:** 2023-01-09

**Authors:** Jing Li, Chang-Yu Qiu, Yue-Jin Tao, Lei Cheng

**Affiliations:** ^1^ Department of Otorhinolaryngology & Clinical Allergy Center, The First Affiliated Hospital, Nanjing Medical University, Nanjing, China; ^2^ Department of Otorhinolaryngology, The Affiliated Jiangning Hospital, Nanjing Medical University, Nanjing, China; ^3^ International Centre for Allergy Research, Nanjing Medical University, Nanjing, China

**Keywords:** chronic rhinosinusitis, nasal polyps, DNA methylation, histone modifications, microRNA, circular RNA, long non-coding RNA

## Abstract

Chronic rhinosinusitis (CRS) has brought a huge socioeconomic burden. However, its mechanism is still elusive, which may involve genetic, environmental and some other factors. Epigenetic analyses have been conducted to explore the mechanisms underlying CRS. Here, we reviewed the fruits in the epigenetic studies on DNA methylation, histone modification, and non-coding RNA regulation. We concluded that the epigenetic research on CRS has made great breakthroughs, especially in the past 5 years and the field of microRNAs. “Epigenetic therapies” are expected to be designed to treat CRS in the future.

## Introduction

Chronic rhinosinusitis (CRS) is one most common otorhinolaryngological disease, characterized by at least 12 weeks of chronic inflammation of the paranasal sinus mucosa (Fokkens et al., 2020). According to its phenotypes, CRS is classified into CRS with nasal polyps (CRSwNP) and without nasal polyps (CRSsNP). CRSwNP can be also subdivided into eosinophilic CRSwNP (ECRSwNP) and non-eosinophilic CRSwNP (non-ECRSwNP) based on the status of eosinophil infiltration (Liu et al., 2020a). The prevalence of CRS in Asia ranges from 2.1% to 28.4% (Chee et al., 2022) and from 4.5% to 12% in North American and European countries (DeConde and Soler, 2016). Genetics, bacterial infection, occupation and environment co-work in the pathogenesis of CRS (Liu et al., 2020a; Fokkens et al., 2020). However, current understanding about CRS mechanisms remains to be expanded.

Waddington (1939) has proposed a term “epigenetic” in 1939 and used “epigenetic landscape” to describe the molecular and biologic mechanisms that transform a genetic trait into a visualized phenotype. Through epigenetic modifications, mainly including DNA methylation, histone modification and non-coding RNA (ncRNA) regulation, a heritable phenotype can be generated without changing DNA sequence. These mechanisms are triggered to boost or suppress gene expression, through which an individual turns susceptible to disease. Epigenetics can explain why monozygotic twins have the same genetic profiles, but different health conditions and disease phenotypes, and this difference might be mediated by acquired factors, such as environmental, nutritional and psychosocial factors (Kaminsky et al., 2009; Hannon et al., 2018). In the past two decades, epigenetic research has unraveled the mechanisms underlying several gene-associated diseases, such as malignant tumors, autoimmune diseases, and asthma (Charras et al., 2020; Garcia-Martinez et al., 2021; Sheikhpour et al., 2021). Among them, malignant tumors have stayed in the research focus, and several epidrugs have come into clinical practice (Egger et al., 2004; Garcia-Martinez et al., 2021). Epigenetic changes may pass down over two generations, as shown by a murine transgenerational asthma model (Jahreis et al., 2018). Scholars have highlighted the significance of epigenetics in the pathogenesis of asthma and other allergic disorders, suggesting a probability of using epidrugs for prevention (Legaki et al., 2022; Potaczek et al., 2022). Epigenetic mechanisms may be initiated to dysregulate transcription factors involved in the activities of T lymphocytes and other immune cells, ultimately resulting in asthma and allergic disease (Liu et al., 2020b). In this light, the dynamic and reversible epigenetic modifications in allergic rhinitis and CRS have been intensely studied, in the hope of finding new therapeutic targets (Yang et al., 2019; Liu et al., 2020b). Latest epigenetic research on allergic rhinitis has been reviewed (Yang et al., 2019; Yu et al., 2021a). Besides, genetic and epigenetic studies of nasal polyps have also been systematically reviewed; the conclusion indicates that epigenetics is involved in biological functions, such as cell cycle, cell proliferation, inflammation, and immune response (Martin et al., 2021).

**FIGURE 1:**
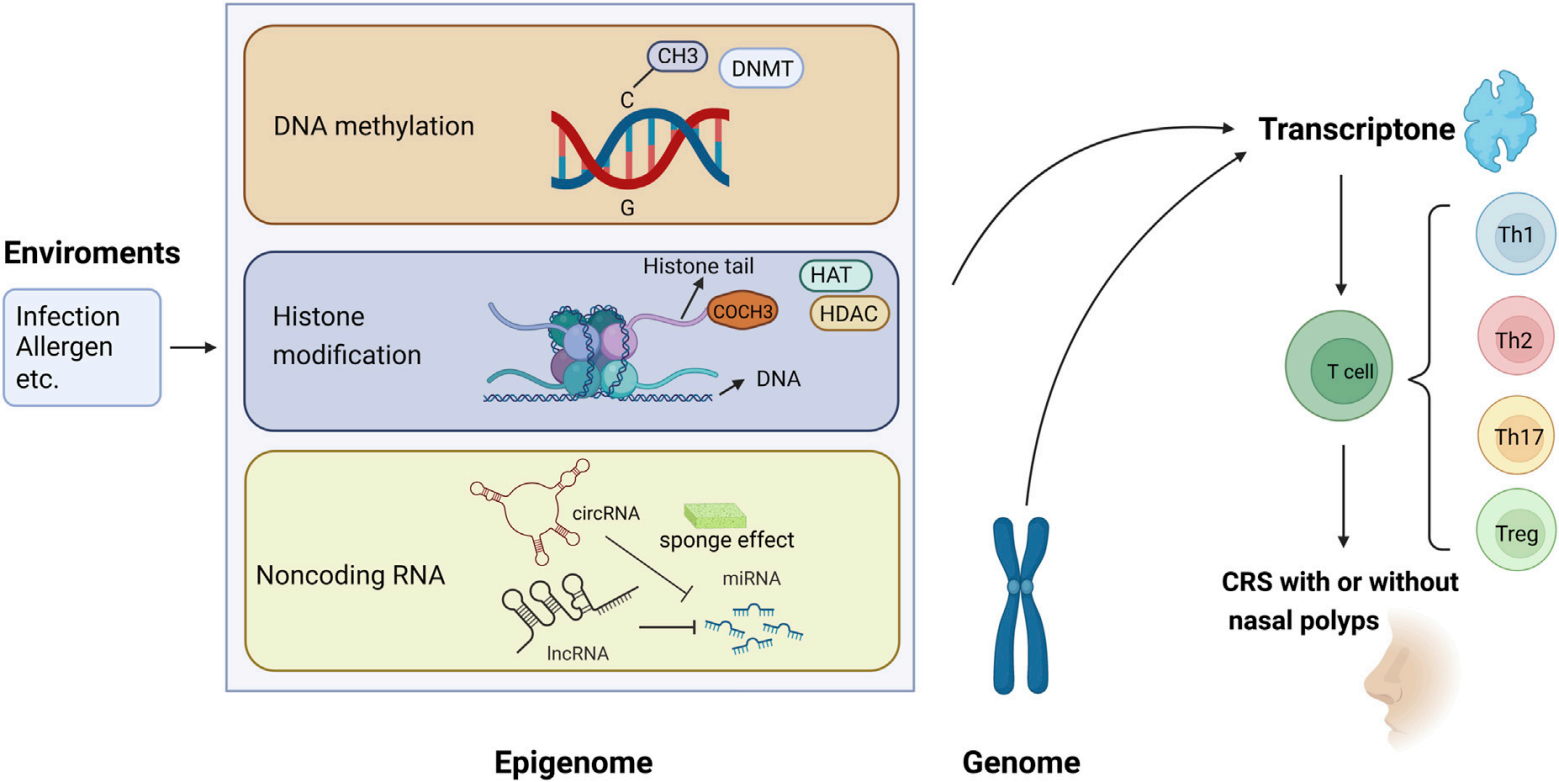
Epigenetic modifications regulate gene expression and contribute to the pathogenesis of chronic rhinosinusitis (CRS) with or without nasal polyps. (1) Methyl group (CH3) on cytosines inactivates gene transcription, which is catalyzed by DNA methyltransferase (DNMT); in contrast, the DNA demethylation contributes to gene expression. (2) When acetyl groups (COCH3) bind to histone tails *via* histone acetyltransferase (HAT), the gene is expressed. Conversely, the removal of the acetyl groups by histone deacetylase (HDAC) turns off the gene expression. (3) circRNA and lncRNA can regulate the 3′-UTR of mRNA through the sponging effect of miRNA, then control the gene expression.

The present review focused on the epigenetic studies about CRS with and without nasal polyps, especially those about DNA methylation, histone modification, and ncRNA regulation (Figure 1). Related literature was search using the following keywords: “epigenetic,” “DNA methylation,” “histone modification,” “non-coding RNA,” “microRNA,” “miRNA,” “circular RNA,” “circRNA,” “long non-coding RNA,” “lncRNA,” “chronic rhinosinusitis,” and “nasal polyps.”

### DNA methylation in CRS

Methylation modification takes place in intergenic regions, gene bodies, and cytosine-phosphate-guanine (CpG) islands, whereas DNA methylation mainly in CpG islands. Gene promoters are a location for gene silencing (Sheikhpour et al., 2021). During DNA methylation, the genes are silenced as the methyl groups are added to the 5′-position of cytosine residues *via* DNA methyltransferases (DNMT). As a representative epigenetic modification, DNA methylation plays an important role in regulating gene transcription (Zhang et al., 2020a). Past decades have seen a burst of studies focusing on DNA methylation in cancers, autoimmune diseases, and various other maladies (Zhang et al., 2020a). To our knowledge, the studies themed with DNA methylation in CRS are limited.

Abnormal DNA methylation, including transcriptional regulation of some immune genes and cytokine production, may be a driver in CRS pathogenesis (Martino et al., 2014). The first related study was conducted in 2011, in which the genome-wide DNA methylation levels are detected in the NP tissues and peripheral blood cells of aspirin-exacerbated respiratory disease (AERD) patients, showing hypermethylation at 332 loci in 296 genes and hypomethylation at 158 loci in 141 genes (Cheong et al., 2011). Furthermore, this study shows that prostaglandin D synthases (PGDS), arachidonate 5-lipoxygenase-activating protein (ALOX5AP), and leukotriene B4 receptor (LTB4R) are hypomethylated, while prostaglandin E synthase (PTGES) is hypermethylated in the arachidonate pathway (Cheong et al., 2011). Kim et al. (2018) have found that the promoter regions of 10 and 30 genes are hypermethylated and hypomethylated in CRSwNP group, respectively. Besides, the top four most hypomethylated genes are keratin 19 (KRT19), nuclear receptor subfamily two group F member 2 (NR2F2), a disintegrin-like and metallopeptidase with thrombospondin type 1 motif 1 (ADAMTS1) and zinc finger protein 222 (ZNF222). Kim et al. (2019) have conducted another study to verify whether the DNA methylation level of CRSwNP varies across endotypes, finding 397 hypermethylated and 399 hypomethylated genes in the ECRSwNP group, as well as 387 hypermethylated and 208 hypomethylated genes in the non-ECRSwNP group, compared to those in the control group. Moreover, four genes exhibit hypermethylation and 19 genes exhibit hypomethylation in the ECRSwNP group, compared to those in the non-ECRSwNP group (Kim et al., 2019). These results show that epigenetic modification is more pronounced in ECRSwNP samples. Besides, frizzled family receptor 5 (FZD5), one most hypomethylated gene in ECRSwNP, has higher mRNA and protein expression levels than those in the non-ECRSwNP group, indicating the engagement of FZD5 in the pathogenesis of ECRSwNP (Kim et al., 2019).

Not all studies hold that CRS involves both hypermethylation and hypomethylation. One study has reported higher methylation levels of 198 genes in the promoter regions in NPs, compared to those in inferior turbinate mucosa samples, and the top four most hypermethylated genes are collagen type XVIII alpha 1 chain (COL18A1), E1A-binding protein p300 (EP300), alpha subunit of the GTP-binding stimulatory protein (GNAS) and Smad ubiquitination regulatory factor 1 (SMURF1). COL18A1, with a higher methylation frequency than the others, has inspired the exploration into the mechanisms of CRSwNP (Zheng et al., 2015). Another report has also shown the DNA methylation levels at all CpG sites are higher in NPs compared with those in inferior turbinate mucosa, with tissue-type plasminogen activator (t-PA, gene name PLAT) as the most obvious (Kidoguchi et al., 2018). The DNA methylation level is negatively correlated to the mRNA expression of PLAT, indicating that PLAT can be a new therapeutic target (Kidoguchi et al., 2018). After adjusting for age, gender, drinking, smoking, asthma and allergic status, hypermethylation at CpG3 and CpG22:23:24 of the thymic stromal lymphopoietin (TSLP) locus is still associated with CRSwNP. It has a positive correlation with olfactory score and unilateral nasal resistance at 75 Pa and 150 Pa, but not with the total nasal resistance or the serum total IgE level (Li et al., 2019a). Therefore, we conclude that DNA hypermethylation at the TSLP locus is a pathogenic factor for CRSwNP and has a potential therapeutic value.

Yao and Xu (2021) have analyzed the TET gene and 5hmC, two markers of DNA demethylation in CRS, finding that high DNA methylation and low DNA demethylation facilitate the progression to CRSwNP, otherwise CRSsNP. This indicates that the DNA methylation or demethylation may decide the phenotype of CRS. Seiberling et al. (2012) have analyzed another two posttranscriptional modifification products, 5-bromocytosine and 5-chlorocytosine, which could lead to DNA methylation and gene silencing. They found that the CRSwNP specimens were significantly higher than those in the control group, suggesting an essential role of DNA methylation and gene silencing in the formation of CRSwNP. Global DNA methylation, DNMT activity, and DNMT expression all increase in CRS tissues, and DNMT expression is positively correlated with Lund-Mackay CT score. Transforming growth factor-β1 (TGF-β1)-induced DNMT expression leads to DNA methylation and epithelial-mesenchymal transition (EMT) *via* p38, c-Jun N-terminal kinase (JNK), Snail, and Slug signaling pathways. It is also observed that inhibition on DNMT suppresses EMT and therefore 5-Aza may be used as a target in CRS therapy (Park et al., 2022). DNA methylation levels at CpG sites 1, 2, and 3 in the IL8 proximal promoter are significantly lower in human nasal epithelial cells (HNEpC) of CRSwNP patients than in those of CRSsNP patients and healthy controls. Furthermore, the percentage of DNA methylation in the CpG3 site has a negative correlation with the levels of tissue-eosinophilic cationic protein and myeloperoxidase, and the methylation of CpG3 interrupts the binding of octamer-binding transcription factor 1 (OCT1) to nuclear factor-kappa B (NF-κB), thus contributing to the pathogenesis of CRSwNP (Li et al., 2019b). Bacterial infection can cause CRS, but the mechanism remains to be fully clarified. Perez-Novo et al. (2013) have found that staphylococcal enterotoxin B stimulation changes the DNA methylation profile of CRSwNP, and both inhibitors of nuclear factor-kappa B kinase subunit beta (IKBKB) and signal transducer and activator of transcription 5B (STAT5B) genes play a key role in T-cell maturation/activation and immune response (Table 1).

**TABLE 1:** DNA methylation involved in chronic rhinosinusitis.

Disease	Upregulated	Downregulated	Pathogenesis	Reference
AERD	PTGES	PGDS, ALOX5AP, LTB4R	Arachidonate pathway	Cheong et al. (2011)
CRS	Overall DNA methylation level		EMT *via* p38, JNK, Snail, and Slug signaling pathways	Park et al. (2022)
CRSwNP	IKBKB, STAT5B,COL18A1, EP300, GNAS, SMURF1, PLAT, TSLP-CpG3/CpG22:23:24. *etc*	KRT19, NR2F2, ADAMTS1, ZNF222, IL8-CpG3, *etc*	1. T-cell maturation/activation; 2. Changing the binding of OCT1 and NF-κB (IL8-CpG3)	Perez-Novo et al. (2013), Zheng et al. (2015), Kidoguchi et al. (2018), Kim et al. (2018), Li et al. (2019a), Li et al. (2019b)
ECRSwNP	397 genes	FZD5, CDH8, PDE4B, WNT2, *etc* (399 genes)	Influence innate epithelial immunity	Kim et al. (2019)

Based on above studies, we conclude that DNA methylation or demethylation can alter the expression of mRNAs and subseqent production of proteins associated with CRS. DNA methylation gives rise to different phenotypes and endotypes of CRS. But previous studies just described this phenomenon, the mechanism of which needs to be further investigated.

### Histone modifications in CRS

Histone modifications, such as histone acetylation, methylation, phosphorylation and ubiquitination, act to repair damaged DNA and maintain nuclear chromatin structure and activity (Alaskhar et al., 2018). Histone acetylation is regulated by histone acetyltransferases (HATs) and histone deacetylases (HDACs). Post-translational modification regulates histone function and recruits T cells and macrophages into airway remodeling, thus evoking allergic symptoms (Alaskhar et al., 2018; Steelant et al., 2019). Compared with DNA methylation, histone modifications have been reported in much fewer studies on CRS. Cho et al. (2013) have observed that trichostatin A (TSA) can suppress TGF-β1-induced myofibroblast differentiation and extracellular matrix (ECM) production by inhibiting HDAC2 and HDAC4 and hyperacetylating histone in NPs. This study provides novel insights into the role of histone in myofibroblast differentiation and ECM production in NPs, making TSA a new candidate in the treatment of CRSwNP. The fourth lysine residue of histone H3 tri-methylated (H3K4me3), which greatly enhances histone methylation, inhibits HNEpC differentiation and promotes the formation of NPs (Yu et al., 2017). Further study shows that both KDM2B (H3K4me3 histone demethylase) and BRG1 (H3K4me3 transcriptional regulator) curb the development of nasal mucosal epithelial inflammation in the phenotype of CRSwNP (Liu et al., 2019a). In summary, histone modifications are common in CRS and should be elucidated in more research.

### ncRNA regulation in CRS

ncRNAs, including microRNAs (miRNAs, miRs), circular RNAs (circRNAs) and long non-coding RNAs (lncRNAs), regulate various biological processes. ncRNAs have been proven active in a body of diseases, such as cancers and autoimmune diseases (Chen et al., 2020; Ali et al., 2021). The research of ncRNAs in CRS, especially miRNAs, has reaped heavy fruits in the past decade.

#### CRS and miRNAs

miRNAs, a group of non-coding single-stranded RNAs with a length of about 22 nucleotides, are encoded by endogenous genes. After transcription, they bind to the 3′-UTR of target mRNAs, thereafter blocking mRNA translation or reducing mRNA stability (Janga and Vallabhaneni, 2011; Chhabra, 2015). Certain miRNAs can directly target to inhibit DNMT, thereby affecting the whole genome methylation (Chhabra, 2015). Studies have shown that the expression levels of miRNA are different between CRSwNP and CRSsNP, even between the endotypes of CRSwNP (Zhang et al., 2014; Ma et al., 2015; Xia et al., 2015). According to their expression, the CRS-related miRNAs are classified into two categories: upregulated miRNAs and downregulated miRNAs.

Here, we first describe the upregulated miRNAs and their mechanisms in CRS pathogenesis. miR-125b is upregulated in CRS, especially in ECRSwNP, suggesting an association between miR-125b and Th2 type inflammation (Zhang et al., 2012; Zhang et al., 2014; Ma et al., 2015; Xia et al., 2015). The mechanism might be that miR-125b induces the production of type I interferon *via* targeting 4E-binding protein 1 (4E-BP1), an initiator of eukaryotic translation, in airway epithelial cells (Zhang et al., 2012). Reports have shown that miR-155 is also upregulated in CRS (Xia et al., 2015), particularly in ECRSwNP (Du et al., 2020). The mechanism of miR-155 in ECRSwNP may involve the increase of inflammatory mediator cyclooxygenase 2 (COX2) and the decrease of anti-inflammatory mediator Src homology-2 domain-containing inositol 5-phosphatase 1 (SHIP1) (Du et al., 2020). Furthermore, the mechanism of glucocorticoids in relieving recurrent CRSwNP is also closely related to the downregulation of miR-155 (Du et al., 2020). miR-19a is upregulated in the peripheral dendritic cells (DCs) of CRSwNP patients and in the HNEpC of CRS patients (Luo et al., 2017; Shin et al., 2020). miR-19a induced by air particulate matter promotes the polarization of proinflammatory M1 macrophages through regulating retinoic acid-related orphan receptor alpha (RORα) expression in human nasal mucosal microenvironment, thus implying the pathogenic effect of environmental factors on CRS (Shin et al., 2020). Besides, upregulating miR-19a can induce IL-4 to suppress the expression of IL-10, whereas the IL-10 level drops significantly in CRSwNP patients (Luo et al., 2017). Additionally, miR-150-5p increases in DCs from the peripheral blood of CRS patients, and it regulates early growth response 2 (EGR2) to promote the formation of CRS *via* the DC-Th axis (Ma et al., 2015; Ma et al., 2018). miR-210-3p and miR-210-5p are also upregulated in the peripheral blood DCs of CRS patients and tissues of CRSwNP patients (Ma et al., 2015; Xuan et al., 2019). However, how these changes arouse diseases has not yet been answered. miR-221 is also upregulated in CRSwNP and may be directly implicated in cell cycle, apoptosis, and inflammation (Bu et al., 2021; Silveira et al., 2021). Bu et al. (2021) have found the expression of miR-449a is higher in non-ECRSwNP samples, compared with that in ECRSwNP samples and healthy controls, and this change involves adenosine monophosphate-activated protein kinase (AMPK), Rap1, and the ErbB-signaling pathway, all regulators in neutrophil activity and neutrophilic inflammation. Meanwhile, Silveira et al. (2021) have also reported a higher level of miR-449a in CRSwNP group, compared with that in control group. Together, we propose that miRNAs may determine the endotypes of CRSwNP *via* targeting mRNAs related to Th1- and Th2-cell differentiation.

In addition, miR-146a (Xia et al., 2015), miR-614 (Shin et al., 2020), and some other DE-miRs are also upregulated in CRS samples, highly through the pathway of mucin-type O-glycan biosynthesis (Cha et al., 2021). Meanwhile, miR-3178, miR-585-3p, miR-3146, miR-320e, miR-142-3p, miR-154, miR-223, miR-205-5p, miR-222-3p, miR-378a-3p, miR-449b-5p, miR-22-3p, miR-21, miR-203a-3p, miR-677, miR-1037, and miR-79 are also overexpressed in CRSwNP samples (Li et al., 2019c; Xuan et al., 2019; Zhang et al., 2020b; Bu et al., 2021; Qing et al., 2021; Silveira et al., 2021; He et al., 2022; Morawska-Kochman et al., 2022). Among them, miR-142-3p may regulate the inflammatory response through the lipopolysaccharide (LPS)-Toll-like receptor (TLR)-tumor necrosis factor-α (TNF-α) signaling pathway (Qing et al., 2021); miR-3146 and miR-320e act in the mucin type O-glycan biosynthesis pathway in CRSwNP by targeting N-acetylgalactosaminyltransferase-like 6 (GALNTL6), N-acetylgalactosaminyltransferase 8 (GALNT8), and alpha-2,3-sialyltransferase 1 (ST3GAL1) genes (Xuan et al., 2019); miR-223 plays in the mitogen-activated protein kinase (MAPK) signaling pathway (Bu et al., 2021); miR-22-3p inhibits vascular endothelial (VE)-cadherin expression to regulate vascular permeability after binding to the specific site in the 3′-UTR of the human VE-cadherin mRNA (Zhang et al., 2020b); the TGF-β1-miR-21-PTEN-Akt axis may propel the EMT of CRSwNP (Li et al., 2019c); miR-677, miR-1037, and miR-79 may post-transcriptionally regulate gene expression in the Hippo signaling pathway (He et al., 2022). Different miRNAs have been observed in different subtypes of CRSwNP. For instance, let-7 and miR-34 are upregulated in non-ECRSwNP (Bu et al., 2021), but miR-205-5p was overexpressed in ECRSwNP (Silveira et al., 2021). Besides, the level of miR-205-5p is positively correlated with IL-5 concentration and eosinophil count in the NPs, and the patients with a higher miR-205-5p level always have a lower favorable 22-item Sino-Nasal Outcome Test (SNOT-22) score and less clinical presentations (Silveira et al., 2021).

The results about miRNA regulation vary with studies. For example, some studies have reported that miR-125b is upregulated (Zhang et al., 2012; Zhang et al., 2014; Ma et al., 2015; Xia et al., 2015), but Xuan et al. (2019) have found that miR-125b-2-3p is downregulated. One shows that let-7 is overexpressed in non-ECRSwNP (Bu et al., 2021), while another shows that let-7a-5p is significantly downregulated *via* the Ras-MAPK pathway in CRSwNP (Zhang et al., 2021). Another controversy is about miR-146a in CRS and CRSwNP, a miRNA that has been proven either upregulated (Xia et al., 2015) or downregulated (Yan et al., 2020; Yu et al., 2021b). Additional analysis reveals that by repressing epidermal growth factor receptor (EGFR), miR-146a modulates mucin-5AC (MUC5AC) induced by human neutrophil elastase hypersecretion, and EGFR is a target gene of miR-146a (Yan et al., 2020). What’s more, miR-146a-5p expression shows no significant difference between healthy subjects and CRSwNP patients (Morawska-Kochman et al., 2022). The reasons for these conflicting results are unclear and need further investigation.

In addition, a large number of downregulated miRNAs have also been found. A study has shown that the expression of 192 miRNA is significantly downregulated, and none are upregulated in the CRSwNP group. Furthermore, its expression displays no statistical difference between ECRSwNP and non-ECRSwNP (Yu et al., 2021b). Recently, Li and Liu (2022) have found 207 lowly expressed miRNAs and constructed 598 miRNA-mRNA pairs in ECRSwNP. The level of miR-124 decreases in CRSwNP, and is negatively correlated with the expression of aryl hydrocarbon receptor (AHR) and TNF-α, suggesting that miR-124 can influence the differentiation of Th1 and Th17 cells by inhibiting STAT1 and STAT3 activation in the presence of protein inhibitor cytokine signaling 5 (SOCS5) (Liu et al., 2018). The expression level of miR-1 is inversely correlated with the level of sinonasal eosinophilia in patients with CRS (Korde et al., 2020). Further murine models have revealed that miR-1 blocks eosinophil recruitment to the airways, relying on P-selectin (SELP), TSLP, eotaxin-3, and thrombopoietin receptor (MPL) in the endothelium (Korde et al., 2020). Another study has identified six downregulated miRNAs in the CRSwNP group, compared with those in the CRSsNP. Among them, miR-4492 exhibits an inverse correlation with IL-10 but not TNF-α, indicating the miR-4492/IL-10 interaction in the Jak/STAT signaling pathway involved in CRSwNP (Li et al., 2019d). Xuan et al. (2019) have found 19 downregulated miRNAs in the tissues of CRSwNP, including miR-32-3p, miR-1299, miR-3196, miR-3924, miR-548e-3p, miR-3184-5p, miR-375, miR-23a-5p, miR-377-5p, miR-574-5p, miR-3149, miR-500a-5p, miR-125b-2-3p, miR-1914-5p, miR-532-3p, miR-612, miR-1298-5p, miR-1226-3p, and miR-668-3p. Kyoto Encyclopedia of Genes and Genomes (KEGG) pathway analysis shows that TGF-β, transient receptor potential (TRP) channels, and the MAPK signaling pathway are significantly enriched in these 19 miRNAs. Among them, miR-500a-5p, miR-532-3p, and miR-548e-3p are critical regulators in the TGF-β signaling pathway in CRSwNP (Xuan et al., 2019). A total of 66 downregulated miRNAs have been detected, and the top three with the most significant changes are miR-192, miR-1022, and miR-4. Gene ontology (GO) and KEGG analyses have showed that these dysregulated miRNAs are involved in multiple pathways, such as the Hippo, the Notch, the ErbB, and the cAMP signaling pathway (He et al., 2022). miR-181b is downregulated in CRS and NF-κB pathway (Xia et al., 2015). In addition, the receiver operating characteristic (ROC) curve analysis has shown the area under the curve (AUC) of miR-145-5p in NPs is .8690, with a *p*-value statistically significant, strongly supporting its involvement in the genesis of CRSwNP (Yu et al., 2021b). Besides, miRNAs are also downregulated although the mechanism yet-to-be explored, such as miR-92a, miR-26b, miR-708-5p, and miR-126-3p in CRS (Ma et al., 2015; Xia et al., 2015), miR-132-3p, miR-27b-3p, miR-17-5p, and miR-125a in CRSwNP (Yu et al., 2021b; Chen et al., 2022; Morawska-Kochman et al., 2022), miR-26a in ECRSwNP (Zhang et al., 2014), and miR-1290 in CRSsNP (Ma et al., 2015) (Table 2).

**TABLE 2:** MicroRNAs involved in chronic rhinosinusitis.

Disease	Upregulated	Downregulated	Pathogenesis	Reference
CRS	miR-125b, miR-125b-5p, miR-155, miR-146a, miR-210-3p, miR-150-5p, miR-19a, miR-614	miR-92a, miR-26b, miR-181b, miR-708-5p, miR-126-3p, miR-146a, miR-1	1. NF-κB pathway (miR-181b); 2. Regulating EGR2 *via* the DC-Th axis (miR-150-5p); 3. Macrophage polarization through the regulation of RORα expression (miR-19a, miR-614); 4. Downregulating the expression of MUC5AC by inhibiting the activation of EGFR, and EGFR is a target gene of miR-146a (miR-146a); 5. Targeting and inhibiting the SELP, CCL26, TSLP, and MPL genes in the endothelium (miR-1); 6. Mucin-type O-glycan biosynthesis was a high-ranked predicted pathway. 7. TGF-β signaling pathway	Ma et al. (2015), Xia et al. (2015), Ma et al. (2018), Korde et al. (2020), Shin et al. (2020), Yan et al. (2020), Cha et al. (2021)
CRSsNP		miR-1290		Ma et al. (2015)
CRSwNP	miR-19a, miR-210-5p, miR-3178, miR-585-3p, miR-3146, miR-320e, miR-21, miR-22-3p, miR-154, miR-221, miR-223, miR-205-5p, miR-221-3p, miR-222-3p, miR-378a-3p, miR-449a, miR-449b-5p, miR-142-3p, miR-203a-3p, miR-677, miR-1037, miR-79	miR124, miR-32-3p, miR-1299, miR-3196, miR-3924, miR-548e-3p, miR-3184-5p, miR-375, miR-23a-5p, miR-377-5p, miR-574-5p, miR-3149, miR-500a-5p, miR-125b-2-3p, miR-1914-5p, miR-532-3p, miR-612, miR-1298-5p, miR-1226-3p, miR-668-3p, miR-4492, let-7a-5p, miR-132-3p, miR-145-5p, miR-146a-5p, miR-27b-3p, miR-17-5p, miR-125a, miR-192, miR-1022, miR-4	1. IL-4 can suppress the IL-10 expression in DCs *via* upregulating miR-19a (miR-19a); 2. LPS-TLR-AHR signaling pathway (miR124); 3. Mucin type O-glycan biosynthesis pathway (miR-3146, miR-320e); 4. TGF-β, TRP channels, and MAPK signaling pathway (miR-548e-3p, miR-500a-5p, miR-532-3p); 5. TGF-β1-miR-21-PTEN-Akt axis (miR-21); 6. Jak/STAT signaling pathway (miR-4492); 7. Regulating vascular permeability through targeting VE-cadherin (miR-22-3p); 8. Mucin-type O-glycan biosynthesis, MAPK signaling pathway, cytokine-cytokine receptor interaction, and Rap1-signaling pathway (miR-223); 9. T2 inflammation (miR-205-5p, miR-221-3p, miR-222-3p); 10. Cell cycle regulation and apoptosis, and to a minor extent, with inflammation (miR-378a-3p, miR-449a, miR-449b-5p); 11. LPS-TLR-TNF-α signaling pathway (miR-142-3p); 12. Ras-MAPK pathway (let-7a-5p); 13. Pro-apoptotic transcripts (miR-203a-3p); 14. MIAT/miR-125a/IRF4 axis (miR-125a); 15. Post-transcriptionally regulate gene expression in the Hippo signaling pathway (miR-677, miR-1037, miR-79)	Luo et al. (2017), Liu et al. (2018), Li et al., (2019c), Li et al. (2019d), Xuan et al. (2019), Zhang et al. (2020b), Bu et al. (2021), Yu et al. (2021b), Qing et al. (2021), Silveira et al. (2021), Zhang et al. (2021), Chen et al. (2022), He et al. (2022), Morawska-Kochman et al. (2022)
ECRSwNP	miR-125b, miR-155	miR-26a	1. Inducing the production of type I interferon *via* targeting 4E-BP1 in airway epithelial cells (miR-125b); 2. Increasing inflammatory mediator COX2 while decreasing anti-inflammatory mediator SHIP1 (miR-155)	Zhang et al. (2012), Du et al. (2020), Li and Liu, (2022)
non-ECRSwNP	let-7, miR-34, miR-449a		AMPK, Rap1, and ErbB-signaling pathway (miR-449a)	Bu et al. (2021)

#### CRS and circRNAs

CircRNAs, structured in a stable and closed continuous loop, act as miRNA sponges. Rich in miRNA-binding sites, circRNAs can increase the expression of target genes by mitigating the inhibitory effect of miRNAs on them (Kulcheski et al., 2016; Chen et al., 2021). Recently, circRNAs have been identified as efficient biomarkers for cancer and allergic rhinitis (Qiu et al., 2021; Rahmati et al., 2021). However, few studies on the performance of circRNAs in CRS are available. The latest research has found 1,081 upregulated and 1,794 downregulated circRNAs in the CRSwNP samples (Yu et al., 2021b). The expression profiles of circ-0031593 and circ-0031594 are significantly higher while those of circ-0109623 and circ-0000736 are lower in the CRSwNP group, compare to the control group. To estimate the function of circ-0031593 in the development of CRSwNP, ROC curve analysis shows that the AUC is .8353, indicating a positive value (Yu et al., 2021b). This research also described the co-expression network in CRSwNP, demonstrating that one circRNA can sponge multiple miRNAs, and one miRNA can be sponged by multiple circRNAs (Yu et al., 2021b). It has been reported that circARRDC3, circ-0008668, circTRIQK, circ-0029853 and circ-01002 are differently expressed in allergic rhinitis (Wang et al., 2021a; Qiu et al., 2021), but their potential roles in CRS need to be further investigated.

#### CRS and lncRNAs

LncRNAs, the other subclass of ncRNAs, are longer than 200 nucleotides. As competing endogenous RNAs (ceRNAs), lncRNAs can also regulate the 3′-UTR of mRNAs through the sponging effect of miRNAs. Recently, accumulating evidence has indicated that lncRNAs regulate various pathological processes, such as cancer and central nervous system disease (Yang et al., 2018; Chen et al., 2020). But up to date, few research has delved into the effect of lncRNAs on CRS. Liu et al. (2019b) have observed 56 upregulated and 209 downregulated lncRNAs in the peripheral blood samples of CRSwNP patients. Among them, lncRNA XLOC-010280 can upregulate the mRNA expression of CCL18 and promote eosinophilic inflammation; while lncRNA RP11-798M19.6, which regulates polypeptide GALNT7 and cell proliferation, is downregulated in patients with CRSwNP (Wang et al., 2016; Liu et al., 2019b). Wang et al. (2020) have identified 312 differentially expressed lncRNAs between the “CRSwNP + asthma” group and the “CRSwNP-alone” group, and further evidence suggests their involvement in arachidonic acid metabolism, fibrinolysis pathway and type 2 cytokines related pathway. Of these lncRNAs, HK3-006 is a hub (Wang et al., 2020). Recently, LINC01198, LINC01094, LINC01798, LINC01829, and LINC01320 have also been identified as CRSwNP associated lncRNAs. Furthermore, LINC01198 promotes the formation of CRSwNP through the sponging effect of miR-6776-5p (Wang et al., 2022). In a recent study, an ECRSwNP-associated lncRNA-miRNA-mRNA ceRNA network has been constructed, composed of 70 lncRNA-miRNA and 598 miRNA-mRNA pairs. This network showcases six hub differently expressed lncRNAs (MIAT, HOTAIRM1, TSPOAP1-AS1, MSC-AS1, LBX2-AS1, and CARD8-AS1), which interact with miRNAs to regulate the expression of mRNAs in ECRSwNP patients. Among these lncRNAs, CARD8-AS1 and MSC-AS1 can act as ceRNA (Li and Liu, 2022). Recently, Chen et al. (2022) have constructed another lncRNA-miRNA-mRNA ceRNA network of CRSwNP, which contains 21 lncRNAs, 8 miRNAs and 8 mRNAs; they proved that the MIAT/miR-125a/IRF4 axis drives the progression of CRSwNP. In addition, the circHIPK3-lncGAS5-miR-495 network charges Th2 differentiation in allergic rhinitis (Zhu et al., 2020).

Although the research on CRS-related lncRNAs is just budding, its results have opened new windows into CRS pathogenesis (Table 3).

**TABLE 3:** lncRNAs and circRNAs involved in chronic rhinosinusitis.

Disease	Upregulated	Downregulated	Pathogenesis	Reference
CRSwNP	lncRNA XLOC_010280, HK3-006, LINC01198, LINC01094, LINC01798, LINC01829, LINC01320. *etc.* hsa-circ-0031593, hsa-circ-0031594. *etc* (1,081 circRNAs)	RP11798M19.6. 1794 circRNAs	1. Regulating CCL18 and inflammation (lncRNA XLOC_010280); 2. Regulating GALNT7 and cell proliferation (RP11798M19.6); 3. Regulating arachidonic acid metabolism, type 2 cytokines related pathway and fibrinolysis pathway (HK3-006); 4. Through sponge miR-6776-5p (LINC01198); 5. MIAT/miR-125a/IRF4 axis; 6. Chemokine signal path and PI3K/AKT signal path	Wang et al. (2016), Wang et al. (2020), Yu et al. (2021b), Chen et al. (2022), Wang et al. (2022)
ECRSwNP	MIAT, HOTAIRM1, TSPOAP1-AS1, MSC-AS1, LBX2-AS1, CARD8-AS1, lncRNA XLOC_010280		1. Affecting the expression of all the DEmRs by interaction with the DEmiRs (MSC-AS1,CARD8-AS1); 2.Regulating CCL18 and eosinophilic inflammation (lncRNA XLOC_010280)	Liu et al. (2019b), Li and Liu (2022)

## Concluding remarks and suggestions for future works

To sum up, the most well-studied epigenetic modification in CRS is miRNA regulation, followed by DNA methylation, whereas the research field of histone modifications, circRNA and lncRNA regulation await further cultivation. The epigenetic research on CRS has risen more than a decade before and made breakthroughs in the past 5 years. In the reported literature, the molecules of epigenetic modification differ with studies, and only a few have been jointly identified by different studies. The reasons may relate with race, age, sex, environment and endotype of CRS. In previous epigenetic studies, the samples are mostly taken from the peripheral blood, and monocytes or DCs are usually isolated (Zhang et al., 2012; Ma et al., 2015; Luo et al., 2017; Pattarayan et al., 2018). As epigenetic changes are tissue- or cell-specific, NP tissue, HNEpC of the inferior turbinate or unciform process mucosa, and extracellular vesicles from nasal lavage fluid have been adopted in recent studies (Steelant et al., 2019; Shin et al., 2020; Cha et al., 2021; Park et al., 2022). Systematic review has also shown growing evidence of the role of exosome miRNAs in the development of CRS (Dzaman and Czerwaty, 2022). Epigenetic modifications happen in a wide spectrum of organs and diseases, so the sensitivity and specificity of epigenetic biomarkers obtained by traditional high-throughput techniques are low. Single cell sequencing technology may overcome this limitation (Ordovas-Montanes et al., 2018). Currently, genome-wide association study (GWAS), epigenome-wide association studies (EWAS), GO, KEGG pathway, and gene expression omnibus (GEO) database are widely applied in studies based on genetics and epigenetics (Li et al., 2019d; Yu et al., 2021b; Chen et al., 2022).

The pathological heterogeneity of CRS results in poor therapeutic responses (Huang et al., 2022). In addition to the epigenetic modifications we reviewed here, recent studies have also identified new genes associated with CRSwNP. Wang et al. (2021b) have found that the expression of stimulator of interferon genes (STING) in ECRSwNP decreases significantly, when compared with that in non-ECRSwNP and control groups, and the potential mechanism might be that the IL-13 signaling is potentiated as the expression of suppressor of cytokine signaling 1 (SOCS1) decreases. Another eight genes (ALOX5AP, BCL2A1, BTK, CYBB, NCF2, HCK, HK3, and MAP1B) have also been identified as indispensable for CRSwNP pathogenesis (Hao et al., 2021; Lan et al., 2021). Some CRSwNP patients manifest poor therapeutic responses when treated with traditional drugs in the past. Recent advances in genetic and epigenetic studies provide new therapeutic targets, which may benefit those with refractory CRS. *In vitro* HNEpC experiments have confirmed that the drugs targeting epigenetic biomarkers can reduce CRS-related inflammatory cytokines and reverse pathological changes, such as EMT (Yu et al., 2017; Liu et al., 2018; Liu et al., 2019a; Korde et al., 2020; Park et al., 2022). Animal experiments have also shown that the inhibitors of DNMTs and HDACs can alleviate allergic symptoms and decrease inflammatory cytokines in allergic rhinitis mice (Wang et al., 2015; Jahreis et al., 2018). Besides, the “epigenetic therapies” have combated some malignancies for nearly 20 years and achieved promising curative effects (Egger et al., 2004). In this effort, the harvests in epigenetic modification research may lay a foundation to design “epigenetic therapies” for CRS in the future.

In general, the present data suggested that previous epigenetic studies on CRS have reaped plentiful fruits, especially in the past 5 years and the field of miRNAs. However, the epigenetic research of CRS is still in its infancy and there is still a long way to go before clinical trials and applications. In the future, we should not only find out more epigenetic molecules, but also their mechanistical pathways. Only in this way can we better design cytological or zoological experiments with epidrugs, and then step forward to clinical practice.

## Glossary

ADAMTS1: A disintegrin-like and metallopeptidase with thrombospondin type 1 motif 1
AERD: Aspirin-exacerbated respiratory disease
AHR: Aryl hydrocarbon receptor
AMPK: Adenosine monophosphate-activated protein kinase
AUC: Area under the curve
ceRNAs: Competing endogenous RNAs
circRNAs: Circular RNAs
COL18A1: Collagen type XVIII alpha 1 chain
COX2: Cyclooxygenase 2
CpG: Cytosine-phosphate-guanine
CRS: Chronic rhinosinusitis
CRSsNP: Chronic rhinosinusitis without nasal polyps
CRSwNP: Chronic rhinosinusitis with nasal polyps
DCs: Dendritic cells
DNMT: DNA methyltransferases
ECM: Extracellular matrix
ECRSwNP: Eosinophilic chronic rhinosinusitis with nasal polyps
EGFR: Epidermal growth factor receptor
EGR2: Early growth response 2
EMT: Epithelial-mesenchymal transition
EP300: E1A-binding protein p300
EWAS: Epigenome-wide association studies
FZD5: Frizzled family receptor 5
GALNTN: N-acetylgalactosaminyltransferase
GALNTL6: N-acetylgalactosaminyltransferase-like 6
GEO: Gene expression omnibus
GNAS: Alpha subunit of the GTP-binding stimulatory protein
GO: Gene ontology
GWAS: Genome-wide association study
HATs: Histone acetyltransferases
HDACs: Histone deacetylases
HNEpC: Human nasal epithelial cells
H3K4me3: Fourth lysine residue of histone H3 tri-methylated
IKBKB: Inhibitor of nuclear factor-kappa B kinase subunit beta
IRF4: Interferon regulatory factor 4
JNK: C-Jun N-terminal kinase
KEGG: Kyoto Encyclopedia of Genes and Genomes
KRT19: Keratin 19
lncRNAs: Long non-coding RNAs
LPS: Lipopolysaccharide
MAPK: Mitogen-activated protein kinase
MIAT: Myocardial infarction associated transcript
miRs: MiRNAs, MicroRNAs
MUC5AC: Mucin-5AC
ncRNAs: Non-coding RNAs
NF-ҡB: Nuclear factor-kappa B
non-ECRSwNP: Non-eosinophilic chronic rhinosinusitis with nasal polyps
NR2F2: Nuclear receptor subfamily 2 group F member 2
OCT1: Octamer-binding transcription factor 1
PGDS: Prostaglandin D synthases
PTGES: Prostaglandin E synthase
ROC: Receiver operating characteristic
RORα: Retinoic acid-related orphan receptor alpha
SELP: P-selectin
SHIP1: Src homology-2 domain-containing inositol 5-phosphatase 1
SMURF1: Smad ubiquitination regulatory factor 1
SNOT-22: 22-item sino-nasal outcome test
SOCS: Suppressor of cytokine signaling
STAT: Signal transducer and activator of transcription
STING: Stimulator of interferon genes
ST3GAL1: Alpha-2,3-sialyltransferase 1
TGF-β: Transforming growth factor-β
TLR: Toll-like receptor
TNF-α: Tumor necrosis factor-α
t-PA: Tissue-type plasminogen activator
TRP: Transient receptor potential
TSA: Trichostatin A
TSLP: Thymic stromal lymphopoietin
VE: Vascular endothelial
ZNF222: Zinc finger protein 222
4E-BP1: 4E-binding protein 1.
